# Smart Coat with a Fully-Embedded Textile Antenna for IoT Applications

**DOI:** 10.3390/s16060938

**Published:** 2016-06-22

**Authors:** Caroline Loss, Ricardo Gonçalves, Catarina Lopes, Pedro Pinho, Rita Salvado

**Affiliations:** 1 FibEnTech Research Unit, Universidade da Beira Interior, Rua Marquês D’Ávila e Bolama, Covilhã 6201-001, Portugal; catarina.lopes@ubi.pt (C.L.); lrbss@ubi.pt (R.S.); 2 Instituto de Telecomunicações, Campus Universitário de Santiago, Aveiro 3810-135, Portugal; rgoncalves@av.it.pt (R.G.); ppinho@deetc.isel.pt (P.P.); 3 Capes Foundation, Ministry of Education of Brazil, Brasília 70040-020, Brazil; 4 Departamento de Eletrónica, Telecomunicações e Informática, Universidade de Aveiro, Campus Universitário de Santiago, Aveiro 3810-135, Portugal; 5 Instituto Superior de Engenharia de Lisboa, Rua Conselheiro Emílio Navarro, Lisboa 1959-009, Portugal

**Keywords:** textile antenna, energy harvesting, smart clothing, wearable devices

## Abstract

The Internet of Things (IoT) scenario is strongly related with the advance of the development of wireless sensor networks (WSN) and radio frequency identification (RFID) systems. Additionally, in the WSN context, for a continuous feed, the integration of textile antennas for energy harvesting into smart clothing is a particularly interesting solution when the replacement of batteries is not easy to practice, such as in wearable devices. This paper presents the *E-Caption: Smart and Sustainable Coat*. It has an embedded dual-band textile antenna for electromagnetic energy harvesting, operating at global system for mobile communication (GSM) 900 and digital cellular system (DCS) 1800 bands. This printed antenna is fully integrated, as its dielectric is the textile material composing the coat itself. The *E-Caption* illustrates the innovative concept of textile antennas that can be manipulated as simple emblems. Seven prototypes of these “emblem” antennas, manufactured by lamination and embroidering techniques are also presented. It is shown that the orientation of the conductive fabric does not influence the performance of the antenna. It is also shown that the direction and number of the stitches in the embroidery may influence the performance of the antenna. Moreover, the comparison of results obtained before and after the integration of the antenna into cloth shows the integration does not affect the behavior of the antenna.

## 1. Introduction

Nowadays, the socio-economic development and lifestyle trends indicate an increasing consumption of technological products and processes, powered by emergent concepts such as the Internet of Things (IoT), where everything is connected in a single network [1]. The development of smart objects for IoT applications, include the capacity of this objects to be identifiable, to communicate and to interact [2]. In this context, wearable technology has been addressed to make the person, mainly through his clothes, able to communicate with, and be part of, this technological network [3].

Wireless communication systems are made up of several electronic components, which, over the years, have been miniaturized and made more flexible, such as batteries, sensors, actuators, data processing units, interconnectors, and antennas [4]. In the systems for on-body applications, the antennas have been challenging, because they are conventionally built on rigid substrates, hindering their efficient and comfortable integration into the garment.

However, embedding antennas into clothing allows expanding the interaction of the user with some electronic devices, making them less invasive and more discrete. Thus, textile antennas that are designed combining the traditional textile materials with new technologies emerge as a potential interface of the human-technology-environment relationship. Textile antennas, thus, become an active part in the wireless communication systems [5,6,7,8,9], aiming applications, such as tracking and navigation [10,11,12], mobile computing, and others [13].

### 1.1. Textile Antennas

For IoT applications, embedding antennas into clothing makes the garments become a smart interface for the interaction between the user and the network. The wearable antennas should be thin, lightweight, of easy or no maintenance, robust, and resistant to washing cycles and usage and, moreover, must be low cost for manufacturing and commercializing [14]. In this way, textile planar antennas, the microstrip patch type, have been proposed for garment applications, because they present all of these characteristics, and also are adaptable to any surface [15]. This type of antenna is usually formed by overlapping conductive (patch and ground plane) and dielectric (substrate) layers [16]. Therefore, the knowledge of the properties of textile materials that are used is crucial, as well as the manufacturing techniques for connecting the layers, such as gluing, seaming, and laminating with adhesive sheets. Furthermore, the microstrip patch antenna radiates perpendicularly to a ground plane, which serves as a shield to the antenna radiation, assuring that the human body absorbs only a very small fraction of the radiation.

### 1.2. Electromagnetic Energy Harvesting

The integration of electronic devices on clothing puts the question about how to feed them. The batteries are an obvious choice, but they are bulky, require frequent replacement or recharging, and their short longevity is an ecological concern of current times. Additionally, the research of self-sustainable wireless devices is a growing challenge in IoT applications [17]. In this context, energy harvesting is a promising solution to consider in the next generation of wireless sensor networks (WSN). Nowadays, radio frequency (RF) energy is currently broadcasted from billions of radio transmitters and, thus, can be collected from the ambient environment or from dedicated sources [18]. Moreover, the advance of technology stimulates the growing number of wireless transmitters, especially in highly populated urban areas, increasing the power density of available RF in the environment [19].

Coherently, in the past years, different proposals of textile antennas for RF energy harvesting have been proposed [19,20,21]. However, only two works [19,20] have outlined the integration of these antennas in smart clothing. In [20] a scheme of jacket with harvester circuit is presented. A triple-band ring textile antenna for RF energy harvesting, operating at GSM900, GSM1800, and WiFi frequencies is also proposed. This antenna was developed with a multilayer configuration and for its conductive parts, the Global EMC shielding fabric with surface resistivity of 0.02 Ω/sq. was used. An unspecified fabric with ε_r_ = 1.23 was used as a substrate support of the antenna. The Kapton^®^ fabric with ε_r_ = 3.4, tanδ = 0.002 and 1 mm of thickness, was used as the dielectric substrate. The predicted antenna efficiencies are 61% at 900 MHz, 54% at 1750 MHz, and 85% at 2450 MHz. In [21] the draft of a coat is presented, where multiple body-worn embroidered textile antennas are proposed to be integrated into a harvester system operating in the 2.45 GHz WLAN band.

## 2. Materials and Methods

**Figure 1:**
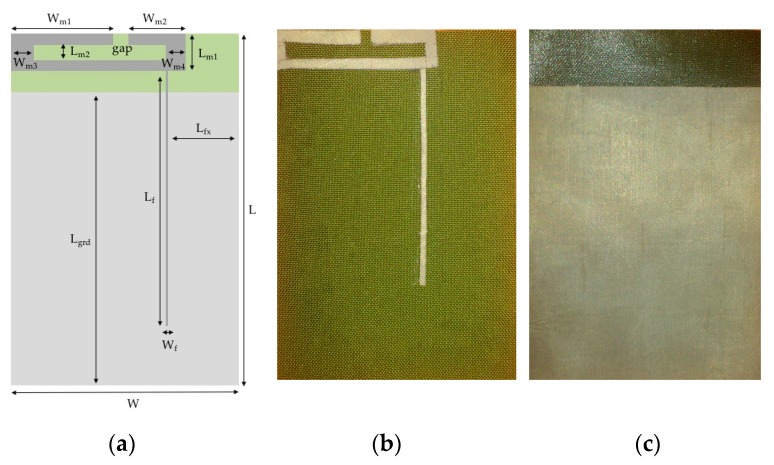
Textile antenna. (**a**) Design of the dual-band antenna; (**b**) Front; and (**c**) Back.

This paper is based on the dual-band textile antenna for GSM900 and DCS1800 frequency bands proposed in [22] that is shown in Figure 1 and which dimensions are given in Table 1 below. The next subsections will analyze the manufacturing process of making this antenna, presenting seven prototypes, made using two different manufacturing techniques: thermal adhesive lamination and embroidering. Further, the integration of the antenna into a smart coat, by fully embedding it into the material constituting the coat, is described. 

### 2.1. Materials

The selection of textile materials for the development of antennas is critical, as discussed in [14]. In this work, all materials used to manufacture the wearable antennas are commercially available and described in the Table 2. For the dielectric substrate, a synthetic fabric with low regain was chosen, in order to minimize the effect of the moisture absorption on lowering the resonance frequency of the antenna.

### 2.2. Manufacturing Techniques

Beyond choosing the textile materials, the construction technique of the antenna is also crucial because the textile materials are highly deformable. The geometrical dimensions of the conductive patch and of the dielectric substrate should remain stable when connecting them, as the mechanical stabilization of both materials is essential to preserve the desired characteristics of the antenna. The geometrical precision of the conductive patch is also critical as the proposed antenna has thin details, as shown in Table 1.

Moreover, the technique to connect the various layers should not affect the electrical properties of the patch, particularly its electrical resistivity. All antennas presented in the following sections were produced assembling the components with the thermal adhesive sheet (JAU Têxteis, Serzedo, Portugal), previously described in the Table 2. The antennas were glued by ironing without steam in a vacuum table. Steam was not used deliberately, especially on materials with copper, to avoid oxidation of the conductive material and the consequent increase of its electrical resistivity.

**Figure 2:**
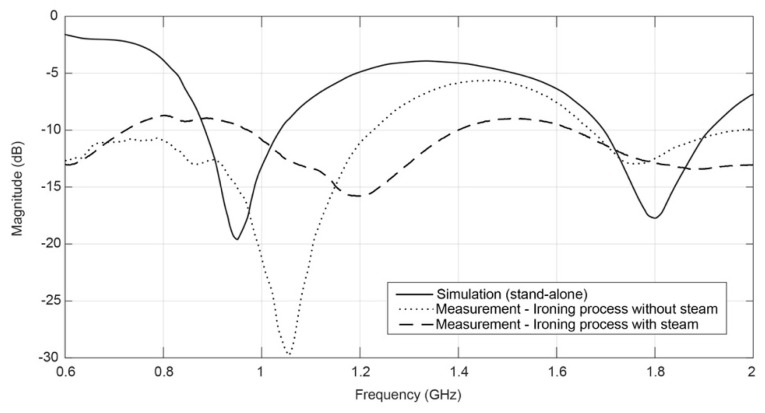
Comparison between ironing processes with, and without, steam.

However, an extra antenna was produced by an ironing process with steam in order to analyse the influence of the steam in the performance of the antenna. Both antennas, with and without steam, were assembled using the same ironing conditions, presented in the Table 3. Additionally, in order to ensure the geometrical accuracy, the patches were cut by an LC6090C CCD (Jinan G. Weike Science & Tecnology Co. Ltd., Jinan, China) laser cutting machine. The obtained results are shown in the Figure 2.

**Figure 3:**
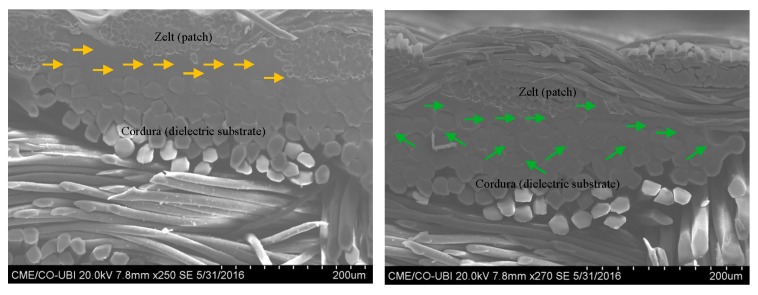
SEM images: cross-section of the antenna assembled (**a**) without steam and (**b**) with steam.

According to the measured results presented on Figure 2, one can see a higher frequency shift in the antenna made using steam in the ironing process. In order to investigate the cause of this shift, the thickness of the antenna was measured, using Kawabata’s Evaluation System (KES) for fabrics (KES-F-3 Compressional Tester). For the antenna without steam, the thickness is 0.62 mm, and for the antenna with steam it is 0.60 mm. This difference can be due to the higher compaction of the materials when steam is applied. As one can see in the scanning electron microscope (SEM) images in Figure 3, in the antenna without steam the adhesive sheet (see yellow arrows) remains at the interface between the conductive and dielectric layers. However, when the steam is applied, the adhesive sheet merges with the textile structure (see green arrows). 

This effect may be responsible for a decrease in conductivity due to the presence of the glue among the conductive yarns. Moreover, the presence of the adhesive sheet into the Cordura fabric (B. W. Wernerfelt Group, Søborg, Danmark) will presumably change, even if slightly, its permittivity. Finally, the shift in frequency may be due to other factors, such as the precision of cutting during the manufacturing process. 

Nevertheless, it is worth noting that it is important to further study the effect of steam in the performance of the antenna, in order to consider and compensate for it in the design of the antenna.

#### 2.2.1. Laminated Antennas

These antennas are made by superposing fabrics and attaching them with a thermal adhesive sheet. The cutting process of the conductive material is critical, as the antenna has very thin lines; for instance, the W_f_ dimension (see Table 1). In order to increase the geometrical accuracy, the patches were cut by an LC6090C CCD laser cutting machine. This procedure also reduces the common fraying effect that appears when cutting thin fabrics with scissors. 

**Figure 4:**
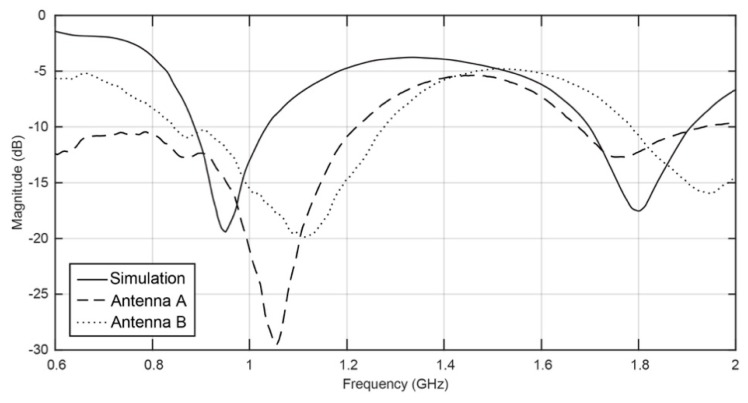
Simulated and measured return loss of laminated antennas.

Two antennas were fabricated with this lamination technique. In order to test the influence of the direction of the structure of the conductive fabric (Zelt) on the performance of the antenna, the patch of antenna A was cut parallel to the warp, and the patch of antenna B was cut at 45° (bias). Figure 4 presents the simulated and measured values of S_11_ parameter of both antennas, measured with a vector network analyser (VNA).

The antennas produced by the lamination technique have shown good results, as the S_11_ parameter shows. As one can see in Figure 4, the measurements match the simulation fairly well, although there is a small shift of the frequency. This shift of frequency might be due to the narrow manufacturing tolerances that exist even when cutting the fabric by laser. Still, the reflection coefficient (S_11_) is low at the operating frequencies, meaning that the antenna presents a good impedance mismatch in both GSM and DCS bands.

**Figure 5:**
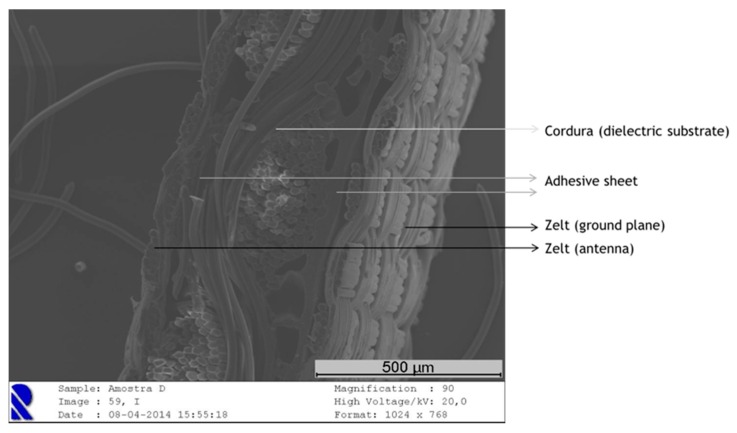
SEM image: cross-section of the textile antenna after assembly with adhesive sheet, without steam.

Thus, with the laminated manufacturing technique, by controlling the ironing conditions, the adhesive may remain at the interface of the conductive and dielectric materials, as one can see in the SEM image in Figure 5, showing the cross-section of a laminated antenna assembled without steam. Therefore, in this antenna, made by ironing without steam, the electrical surface resistance of the patch and the relative permittivity of the substrate are not significantly changed. This observation is corroborated by the S_11_ measurements previously showed in the Figure 4.

#### 2.2.2. Embroidered Antennas

Embroidering is a promising method in terms of repeatability and mass-manufacturing [24], as the embroidered antennas do not need a cutting or lamination process, thus reducing the production costs. For this reason, several embroidered antennas have been proposed; for instance, spiral antennas [25,26], RFID tags [27,28], and antennas without a ground plane [29,30,31]. This paper explores the embroidering technique to produce antennas that can be easily applied in clothing as an emblem. This way the embroidering technique might enlarge the dissemination of the textile antennas into clothing. 

As the antenna considered in this paper is a printed monopole antenna which requires a ground plane, the manufacturing process has to be adapted in order to eliminate the short cuts caused by the embroidering technique in both sides of the dielectric material. Therefore, the construction technique of the embroidered antenna was: firstly, embroidering the patch in a thin textile; secondly, cut the embroidery; and, finally, attach the embroidery to the dielectric substrate using the thermal adhesive sheet. This process is the same one used to produce the traditional emblems for cloth customization. 

Five antennas were developed with this technique, using a SWF MA-6 automatic embroidering machine. The patches were embroidered in the Atlantic fabric (B. W. Wernerfelt Group, Søborg, Danmark) using Silverpam yarn (Tibtech Innovations, Pierre Mauroy, France) (see Table 2). The parameters of the embroidery are described in Table 4. The orientation of the stitch was considered by performing stitches along four different directions for antennas 1, 3, 4, and 5. The number of stiches was considered by varying the float of the stitch for antennas 2 and 3. To avoid differences in the fringe effect on the feed line (W_f_ × L_f_) due the different directions of the stitches, all antennas have feed lines embroidered with a horizontal step stitch.

**Figure 6:**
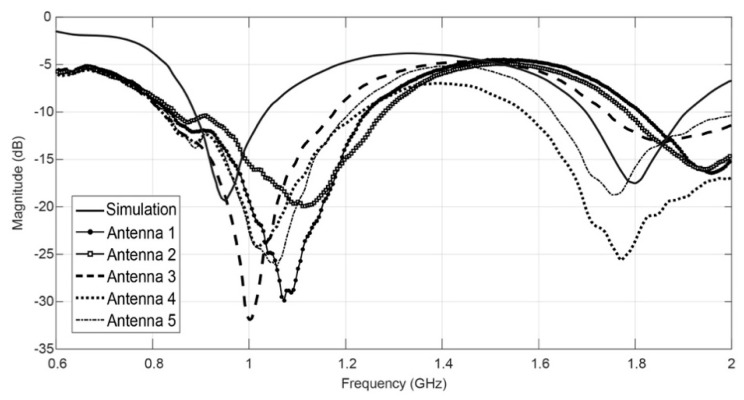
Simulated and measured return loss of embroidered antennas.

Figure 6 shows the simulated and measured values of the S_11_ parameter of these antennas, measured with a VNA. It is clean that the measurements match closely to the simulations. Where antenna 3 is the one with the best match of the reflection coefficient.

The antenna 3 presents the closest result to the simulation line. This can be due to the fact the embroidery stitch direction is parallel of the feed line, homogenizing the current flow [24]. Antennas 4 and 5 present very similar behavior that might indicate the angle of the diagonal direction is not influencing it. Coherently, antenna 1 shows the higher shift of the frequency that can be due to the fact the direction of the embroidery stitch is perpendicular to the feed line. Additionally, antenna 2 was made using a vertical stitch, as was antenna 3, having however, a higher number of stitches. This makes the current flow less continuous, due the constant breaks and higher number of air gaps in the embroidery [15], reducing the conductivity of the patch, which may explain the difference between the magnitudes of the return loss of antennas 3 and 2.

### 2.3. Integration into Clothing

When developing smart textile products, bringing technologies to the consumer in an acceptable and desirable format is a challenge. Some authors have been integrating textile antennas in products in a pleasing way, for instance, as wearable antennas for commercial advertisement proposes, dissimulated in brand names and logotypes [32,33]. However, until now, the microstrip patch textile antennas have been built ex situ and then posteriorly integrated in the lining of the garment or into pockets or simply glued to the cloth. 

This paper proposes an innovative solution that presents the first antenna prototype manufactured directly in the clothing as it is made with the same textile materials composing the cloth. This cloth is a smart coat for electromagnetic harvesting—the *E-Caption: Smart and Sustainable Coat*, in which the antenna has a substrate that is continuous and was cut according to the pattern-making of the coat, thus being part of it. 

**Figure 7:**
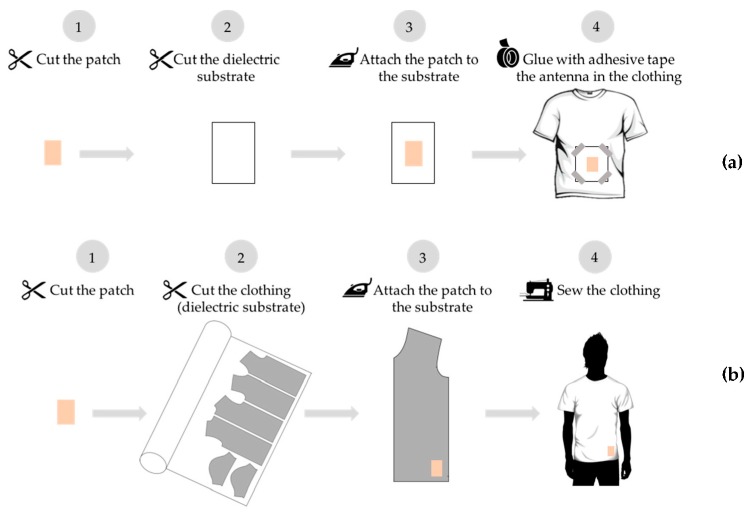
Comparison of techniques of integration of antennas into cloth (**a**) typical integration and (**b**) “emblem” approach.

This innovative solution to integrate antennas into cloths is illustrated in Figure 7. The antenna is integrated into the clothing by manipulating it as a simple emblem. In the future, the antennas can be incorporated into patterns and drawings, mixing conductive and non-conductive embroideries, creating fashionable emblems that function as antennas. These “emblem” antennas may be accessible to the end user for customization of smart cloth, for several applications.

#### E-Caption: Smart and Sustainable Coat

The integration of textile antennas for energy harvesting into smart clothing emerges as a particularly interesting solution when the replacement of batteries is not easy to practice, such as in wearable devices. A fully-embedded antenna in clothing contributes for the integration of electronic devices in less obtrusive ways, improving the good aesthetic and the technical design, making the garment more comfortable and desirable to the final consumer. This might enhance niche markets where form and function work together in order to create new attractive textile products that can assist the user in many aspects of their daily routine.

**Figure 8:**
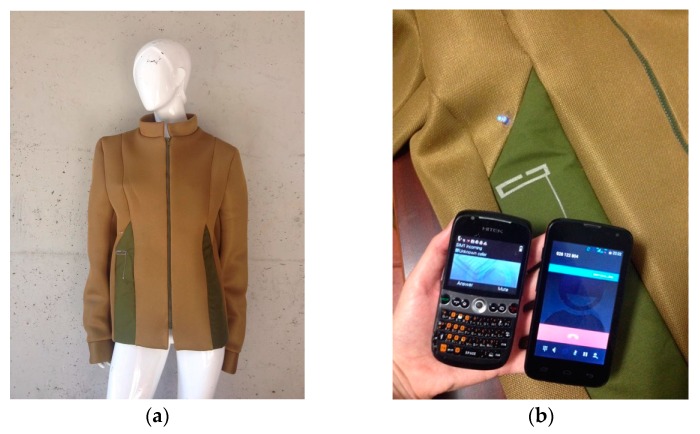
*E-Caption: Smart and Sustainable Coat*. (**a**) Design of the coat and (**b**) integrated antenna, in detail.

The *E-Caption: Smart and Sustainable Coat* was developed combining these concepts, integrating antenna A produced by the lamination manufacturing technique presented in previous sections. It is the first prototype of a smart coat with a printed monopole antenna fully integrated, as its dielectric is the textile material composing the coat itself. The coat is made of Cordura and of a 3D fabric (Reference 3003—3D fabric, from LMA—Leandro Manuel Araújo, Ltda.). Figure 8 shows the *E-Caption* coat with the textile antenna for RF energy harvesting. 

In the past years, some authors have been analyzing the influence of the human body on the performance of textile antennas [34,35]. However, no one has analyzed the influence of its integration on clothing on its performance. Therefore, the analysis of the integration of antennas and the evaluation of their behavior after integration into clothing are discussed in Section 3.

## 3. Results

**Figure 9:**
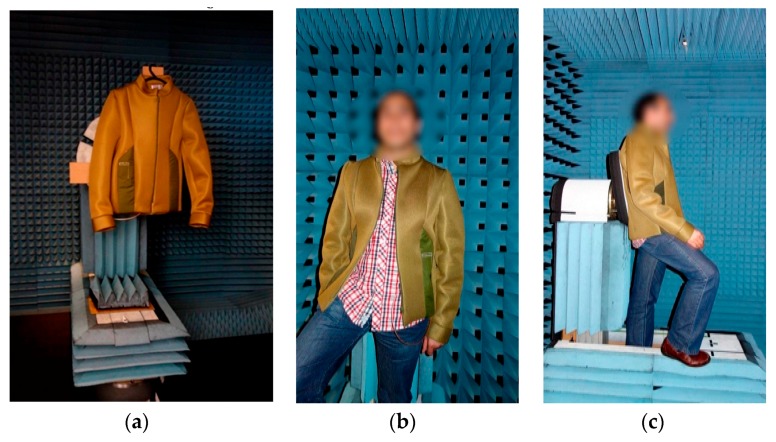
Performance of the antenna in the anechoic chamber (**a**) in free space and (**b**,**c**) on-body measurements.

The performance of the antenna of the *E-Caption: Smart and Sustainable Coat* was tested in the anechoic chamber, as shown in Figure 9. 

**Figure 10:**
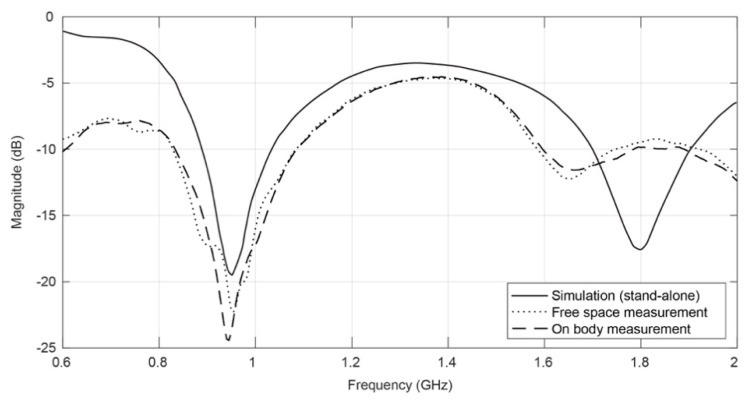
Simulated and measured return loss, before/after the integration on clothing.

Figure 10 presents the variation in the S_11_ parameter obtained through numerical simulation and measured in free space, before and after the integration into the smart coat. It is possible to see the agreement between the simulated and measured values even in the on-body measurements. The textile antenna presents an operating frequency range capable of completely covering the GSM900 (880–960 MHz) and the DCS1800 (1710–1880 MHz).

**Figure 11:**
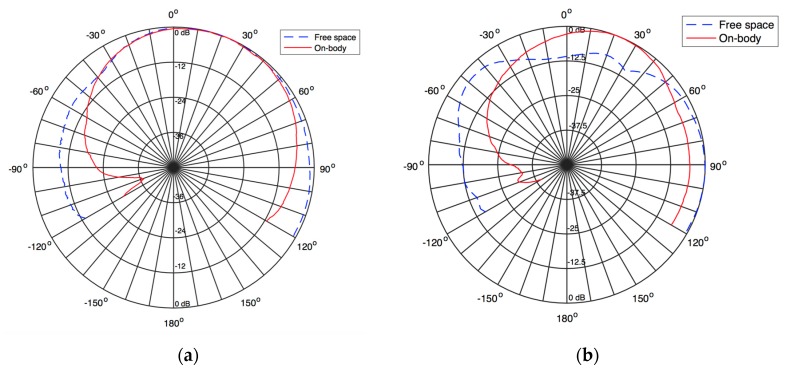
Measured radiation pattern of the textile antenna into the coat at (**a**) 900 MHz and (**b**) 1800 MHz.

Even after integrated into clothing, the radiation pattern of the antenna is clearly omnidirectional. The Figure 11 shows the radiation pattern of the antenna fully integrated into the smart coat structure and also measured on-body. The results depicted in Figure 11 correspond to the XZ plane. This is the only measurable plane (see Figure 9), due to the configuration and placement of the antenna on the coat. Nevertheless, it is the most relevant plane in order to evaluate the omnidirectional characteristic of the antenna. 

Moreover, given the position of the antenna on the coat, previously shown in Figure 8, it is clear that the direction at which the antenna will present less influence from the coat or from the person occurs at nearly 30° in the broadside direction. This is confirmed by the results depicted in Figure 11.

According to the results presented in Figure 11, one may conclude that, as expected, the mass of the coat and mainly of the person influence the radiation characteristic of the antenna. In the measurement of the empty jacket, when the coat places between the probe antenna and the test antenna, around 160°, there is a reduction in the gain of the antenna, which is due to the presence of a large dielectric body, that is, the coat. Nevertheless, the antenna shows a nearly omnidirectional pattern. 

The on-body antenna performs differently. Since the human body is conductive, it absorbs and reflects radiofrequency waves. The results on Figure 11 show that when the body is behind the test antenna, at 30° broadside, a slight increase in gain is measured. However, when the body is between the probe and the test antenna, at 160°, it will absorb a high amount of radiation and will reflect the rest in the opposite direction, shielding the test antenna and, thus, create a null of radiation at this point. This happens for both frequencies, being clearer at 1800 MHz.

## 4. Discussion

In the future, garments will not only communicate social conditions or protect the human body against the extremes of nature, but will also provide information and communication tools. Clothes are becoming able to communicate wirelessly without the need of large and expensive equipment. This is possible because textile technologies can produce new types of sensors and antennas that are so small, flexible, and inexpensive that they can be applied in different types of clothing, shoes, and accessories.

The effective integration of wearable systems contributes to the advance of the IoT. The innovative concept of producing textile antennas to integrate into clothing by simply manipulating it as an “emblem” may improve the usage of the wearable technologies. In the future, the wearable antennas can be incorporated into textile patterns and drawings, creating fashionable antennas. These “emblem” antennas may be easily acceded by the end user, for customization of smart cloth for several applications. 

The *E-Caption: Smart and Sustainable Coat* is the first prototype of this concept, integrating an “emblem” antenna capable of completely covering GSM900 (880–960 MHz) and the DCS1800 (1710–1880 MHz) bands, for IoT applications. In this context, the integration of textile antennas for energy harvesting into smart clothing can be a solution for recharging wearable devices, such as low-power electronics and WBSN.

## 5. Conclusions

Embedding antennas in clothing contributes for the advance of the integration of electronic devices in less obtrusive way making the smart clothes more comfortable. In the *E-caption*, the antenna is manufactured directly on the clothing, having a continuous dielectric substrate made with the textile materials composing the coat. Therefore, a continuous substrate of the antenna does not influence its performance. Moreover, the presented results show that, despite the masses of the coat and of the body influencing the radiation characteristic of the integrated antenna, it still shows a nearly omnidirectional pattern.

This work shows that “emblem” antennas, including the ones having ground planes, may be manufactured by lamination and embroidering techniques. When laminating, the ironing process without steam seems to be preferable as it better preserves the electromagnetic performance of the materials. Additionally, this work shows the orientation of the conductive fabric used for the patch is not influencing the performance of the laminated antenna. In addition, it shows the direction and number of the stitches in the embroidery may contribute to increasing the conductivity of some elements, thus improving the performance.

Other techniques to produce “emblem” antennas may be considered in the future, for instance, transfer, screen printing, and inkjet methods. Finally, this innovative concept of textile antennas for energy harvesting might open new horizons in the clothing development and in sustainable communication.

## Figures and Tables

**Table 1:** Dimensions of the textile antenna.

Parameter	Dimension (mm)
L, L_gnd_, L_f_, L_fx_	120, 100, 78, 30
L_m1_, L_m2_, gap, W	12, 5, 3.1, 80
W_f_, W_m1_, W_m2_, W_m3_, W_m4_	1.5, 31, 21, 8, 4

**Table 2:** Characteristics of the textile materials used to develop wearable antennas.

**Dielectric Materials**	**Fabric**	**Mass per Unit Surface (g/m^2^)**	**Composition**	**Finish**	**Thickness (mm)**	**ε_r_**	**tanδ**
	Cordura^®^ Light	280	100% PA 6.6	Polyurethane coated	0.5	1.9	0.0098
**Conductive Materials**	**Fabric**	**Mass per Unit Surface (g/m^2^)**	**Composition**	**Finish**	**Thickness (mm)**	**Conductivity (S/m)**	
	Zelt	55.87	100% Polyamide	Copper and tin plated	0.06	1.75105	
	**Yarn**	**Linear Mass (dtex) ^1^**	**Composition**	**Finish**	**-**	**Conductivity (S/m)**	
	Silverpam	250	100% Polyamide	Silver plated	-	0.005	
**Other Materials**	**Fabric**	**Mass per Unit Surface (g/m^2^)**	**Composition**	**Finish**	**Thickness (mm)**		
	Atlantic	120	100% Polyester	Oil + water repellent	0.3		
	**Adhesive Sheet Type**	**Mass per Unit Surface (g/m^2^)**	**Composition**	**-**	**Thickness (mm)**		
	Fixorete Losango	0.28	100% Polyamide	-	0.01		

^1^ *Tex* is the unit of the International System of Units used to characterize the linear mass of fibers and yarns. *Tex* is defined as the mass in grams per 1000 m. The subunit *decitex* (dtex) is the mass in grams per 10,000 m [23].

**Table 3:** Ironing conditions.

Temperature (°C)	Pressure (bar)	Time (s)
200	10	12 (6 for patch + 6 for ground plane)

**Table 4:** Parameters of the embroidered antennas.

Antenna	1	2	3	4	5
**Description of Stitch**	Horizontal step stitch	Satin with vertical step	Vertical step stitch, with horizontal step	Diagonal step stitch (direction: 152°/quadrant 2), with horizontal step	Diagonal step stitch (direction: 30°/quadrant 1), with horizontal step
**Draft of Stitch**	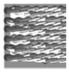	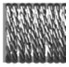	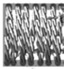	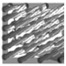	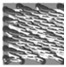
**Number of Stitches**	1255	2084	1378	1360	1361
**Yarn Consumption (g)**	0.27	0.39	0.22	0.23	0.27
**Yarn Consumption per Embroidered Area (g/m^2^)**	489	706	398	416	489
